# How bumblebees manage conflicting information seen on arrival and departure from flowers

**DOI:** 10.1007/s10071-024-01926-x

**Published:** 2025-02-05

**Authors:** Marie-Geneviève Guiraud, HaDi MaBouDi, Joe Woodgate, Olivia K. Bates, Oscar Ramos Rodriguez, Vince Gallo, Andrew B. Barron

**Affiliations:** 1 School of Biological and Behavioural Sciences, Queen Mary University of London, Mile End Road, London, UK; 2 School of Natural Sciences, Macquarie University, Sydney, NSW Australia; 3 School of Biosciences, University of Sheffield, Sheffield, UK; 4 Department of Ecology and Evolution, University of Lausanne, Lausanne, Switzerland

**Keywords:** Active vision, *Bombus terrestris*, Insect cognition, Cognitive visual engram, Visual learning

## Abstract

Bees are flexible and adaptive learners, capable of learning stimuli seen on arrival and at departure from flowers where they have fed. This gives bees the potential to learn all information associated with a feeding event, but it also presents the challenge of managing information that is irrelevant, inconsistent, or conflicting. Here, we examined how presenting bumblebees with conflicting visual information before and after feeding influenced their learning rate and what they learned. Bees were trained to feeder stations mounted in front of a computer monitor. Visual stimuli were displayed behind each feeder station on the monitor. Positively reinforced stimuli (CS +) marked feeders offering sucrose solution. Negatively reinforced stimuli (CS−) marked feeders offering quinine solution. While alighted at the feeder station the stimuli were likely not visible to the bee. The “constant stimulus” training group saw the same stimulus throughout. For the “switched stimulus” training group, the CS + changed to the CS− during feeding. Learning was slower in the “switched stimulus” training group compared to the constant stimulus” group, but the training groups did not differ in their learning performance or the extent to which they generalised their learning. The information conflict in the “switched stimulus” group did not interfere with what had been learned. Differences between the “switched” and “constant stimulus” groups were greater for bees trained on a horizontal CS + than a vertical CS + suggesting bees differ in their processing of vertically and horizontally oriented stimuli. We discuss how bumblebees might resolve this type of information conflict so effectively, drawing on the known neurobiology of their visual learning system.

**Supplementary Information:**

The online version contains supplementary material available at 10.1007/s10071-024-01926-x.

## Introduction

Bees are excellent learners. In nature, their ability to successfully forage relies on their capacity to identify, memorise and return to high quality flowers (Grant 1950). In simple associative paradigms, just three pairings of an odour (Menzel 1999, 2001; Giurfa and Sandoz 2012) or colour (Avarguès-Weber and Giurfa 2014; Muth et al. 2015) with sucrose solution reward is sufficient to establish a lifelong memory in a foraging honey bee. In a classical associative task, the conditioned stimulus (CS) precedes and overlaps with the unconditioned stimulus (US) such that bees learn a tight temporal relationship with the conditioned stimulus predicting the unconditioned stimulus (Menzel 1993; Hammer and Menzel 1995). Bees can learn much more than just this temporal contingency, however. Bees can generalise learned relationships (Giurfa et al. 2001; Bernard et al. 2006), they are capable of trace conditioning (Menzel 2001; Szyszka et al. 2011; Paoli et al. 2023), where there is a gap between the presentation of the CS and US. They can learn conditioned stimuli presented after the US (Menzel 2001; Hussaini et al. 2007) and are capable of latent learning where there is no explicit reinforcement (Menzel et al. 1993; Wystrach 2023). All of these are considered cognitive forms of learning. They give bees great flexibility and capacity to recognise and learn relationships between relevant stimuli, but this flexibility also presents a cognitive challenge. Some relationships between CS and US could be inconsistent, or even contradictory and these could interfere with a bee learning the most useful relationships between CS and US (Menzel 2001, Giurfa and Sandoz 2012). In this study, we examined how inconsistent information affected learning in bumblebees to assess how well an insect brain can manage information conflict.

Classical associative learning is typically explained by Hebbian processes and spike-timing dependent neuroplasticity (Hebbian mechanisms: Caporale and Dan 2008, Johansen et al. 2014, bees neurobiological support: Rath et al. 2011, Galizia 2014). Simply put, the connection between neural circuits for the CS and the conditioned response is modified by the co-activated US. In insects, there are several loci for this mechanism of learning, including the antennal lobes and the mushroom bodies (Galizia 2014). Other types of learning are considered more complex because something more than this simple mechanism of learning is needed to explain them. In backward conditioning a persistent engram exists in the brain (Hall 1984). Turn-back-and-look behaviour (Lehrer 1991, 1993), where bees reorient towards the flower and visualise it as they depart, could be supported by backward conditioning. In traditional forward conditioning, the CS seen during departure doesn’t interfere with what is learned. Simple conditioning would not produce the results we are seeing here as there is an engram of what is seen both before and after feeding (e.g. during the conflict test, bees have a tendency to prefer the intermediate stimuli over the negative stimuli for instance). In trace conditioning there is a temporal gap between the presentation of the CS and US, which also requires some form of enduring neural engram of the CS that persists beyond the presentation of the CS such that it can be related to the later US. Such engrams have been identified in the brains of insects (Menzel 2001; Menzel and Giurfa 2001; Perisse and Waddell 2011).

Lehrer (1991, 1993) provided an early and influential demonstration of cognitive flexibility in honey bee visual learning mechanisms while questioning the efficiency of CS before and after US. Lehrer noticed that upon departing a flower on which a bee had just fed, often the bee would pause in flight and “turn back and look at the flower” (Lehrer 1991, 1993). This motivated Lehrer to study whether bees were learning the features of a flower on approach or departure or both. By manipulating stimuli seen on arrival and departure from the flower, Lehrer was able to show that bees could learn stimuli seen on both arrival and departure from a rewarded flower (Lehrer 1993). If stimuli seen on arrival and departure where inconsistent, then bees preferred the stimulus seen on arrival over the stimulus seen on departure (Lehrer 1993). In classic associative learning theory, a CS that comes after the US is typically not learned since it is not predictive of the occurrence of the US. And yet, bees demonstrate a specific behaviour—the turn back and look—at a feeder on departure and learn features of a feeder during this behaviour. This form of learning could either be a form of secondary reinforcement or latent learning (Menzel 2001). Secondary reinforcement would assume that the feeder station and/or feeder location has become a reinforcer following pairing with food reward, in which case the feeder could now act as a conditioned reinforcer for any view directed at the feeder. Latent learning is simply learning with no explicit reinforcer and is presumed to be important for many forms of spatial learning.

Given that bees can learn stimuli that both precede and succeed a food reward, our objective here was to study how conflicting information presented before and after feeding influenced the speed of learning and what bees learned to understand the robustness and mechanisms underlying such cognitive feat. Lehrer investigated if honeybees can learn stimuli when the timing of learning is manipulated in an absolute conditioning paradigm throughout different conditions. Lehrer asked if bees could learn two CS + with one on approach and one during departure. This can cognitively be done using an additive process or generalisation. Meanwhile, our work builds on it and investigates how bumblebees resolve conflicting visual information in a discriminative conditioning paradigm. We introduced a conflict, so the food is associated with both a CS + and a CS−. Moreover, contrarily to Lehrer where setups are in non-controlled conditions (outside) with only a handful of bees, we used controlled laboratory conditions with a more robust dataset. Bees were trained to feed from Perspex cubes mounted in front of digital displays that allowed stimuli to be instantly changed. While feeding, bees would likely not see the stimuli, and, with this system, we could precisely change the stimuli bees saw on arrival and upon departure from the feeder. We used a discriminant learning paradigm in which CS + was rewarded with sugar solution and CS− was punished with quinine solution. We compared the learning of bees that saw a consistent CS+ on arrival and departure from a sucrose feeder with those that experienced the CS + on arrival but the CS− on departure from the feeder. Bees were subsequently subjected to three unrewarding tests. A learning test, a conflict test and a generalisation test. The learning test was similar to training, the conflict test investigated how bees responded in presence of intermediate visual stimuli and the original stimuli, the generalisation test looked at the consequences of the visual generalisation.

## Material and methods

**Fig. 1:**
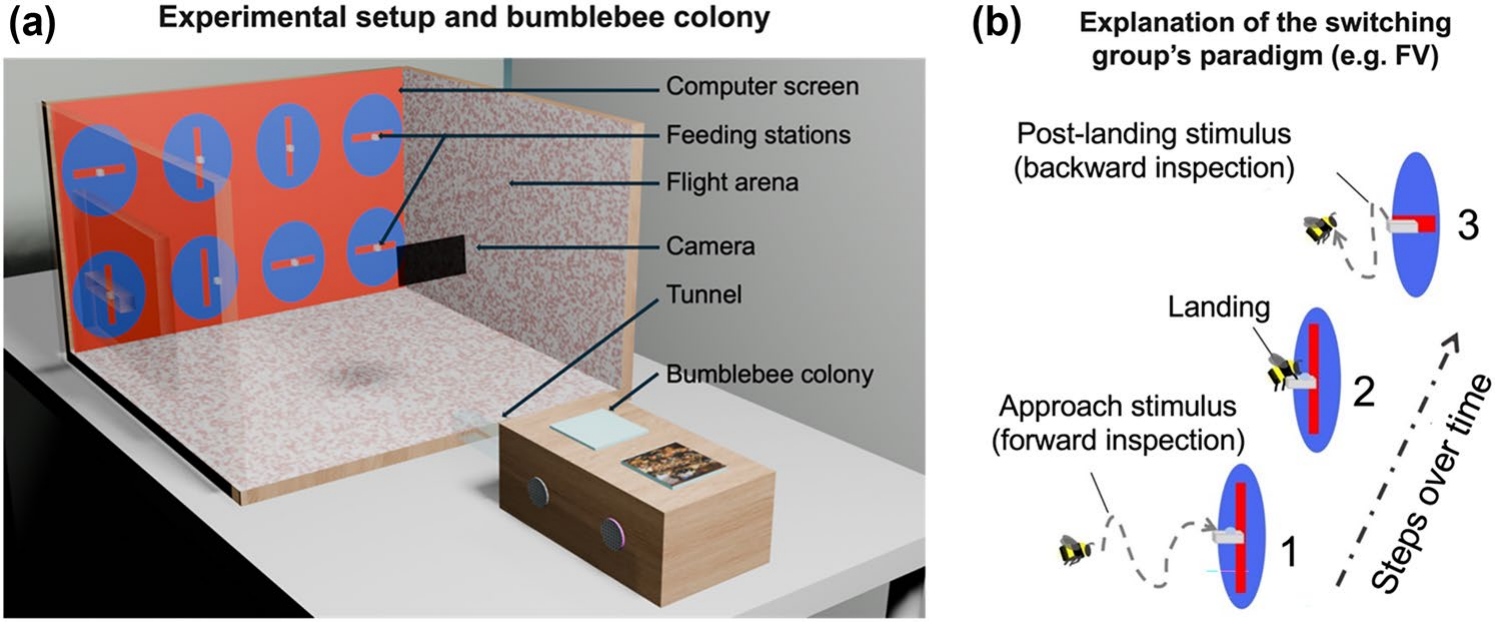
Experimental setup and switching condition. **a** The computer screen at the back of the flight arena displayed eight stimuli, each of which had a feeding station at its centre. Four stimuli provided a sucrose solution (rewarding) and the other four provided quinine solution (punishing). Across training, the location of rewarding stimuli and punishing stimuli changed pseudorandomly. Bees in the constant stimuli groups were exposed to the same rewarded stimulus on approach and post-landing (“constant horizontal stimuli” and “constant vertical stimuli” groups, referred to as CH and CV). Bees in the switching stimuli groups saw different approach and post-landing stimuli (“switching horizontal stimuli” and “switching vertical stimuli” groups, referred to as SH and SV). Example shown here **b** Switching vertical group

Bumblebees (*Bombus terrestris audax*) from seven colonies provided by BIOBEST (Biobest Belgium N.V., Westerlo, Belgium) were used. Each colony was housed in a wooden nest box (28 cm L × 16 cm W × 11 cm H). The nest box was connected to a Perspex tunnel leading to a flight arena (60 cm L × 60 cm W × 40 cm H). Within the flight arena, workers could freely forage for 30% sucrose solution (w/w) from eight transparent feeding cubes (rectangular cuboids to be exact, with the following measures 1.5cm^2^ 0.8 cm H, with a hole 0.6 cm ⌀ and 0.3 cm deep). These feeding stations were fixed vertically to a transparent Perspex wall in front of a computer screen displaying eight blue circles set against a red environment (Fig. 1a). The walls of the flight arena were covered with a laminated pink and white Gaussian dot pattern to provide optic flow for the bees and create contrast between the bee body and the background for video tracking. The arena was illuminated using high-frequency fluorescent lighting (TMS 24F lamps with HF-B 236 TLD ballasts, Phillips, Netherland and fitted with Activa daylight fluorescent tubes, Osram, Germany). Both lights operated at a frequency of approximately ~ 42 kHz. The high-resolution LCD monitors (Acer Predator GN246HLB) employed to display the visual stimuli boasted a refresh rate of 144 Hz significantly suppressing the flicker fusion frequency known for bees (Srinivasan and Lehrer 1984; Skorupski and Chittka 2010). Flight trajectories of bees were recorded by an iPhone camera (iPhone 6, Apple) placed at the rear of the arena, filming at 120 frame per second (fps). Lehrer (1993) used a binary choice apparatus. Our approach used a multiple-choice apparatus to provide more natural foraging environment for bees. Previous work (Chandra et al. 1998), shown that multiple-choice paradigms consistently yield more precise behavioural results, and faster learning rate (Guiraud et al. 2022).

The small volume of sugar solution (10 μL) deposited onto each Perspex cube, was well under the crop capacity of bumblebees, which encouraged bees to visit multiple feeders during a single foraging trip. Workers successfully using the feeders were marked with coloured number tags (Opalithplättchen, Warnholz & Bienenvoigt, Ellerau, Germany).

### Stimuli

Stimuli were generated and displayed on the monitor using custom MATLAB (Mathworks) code in conjunction with the PsychToolbox (Brainard 1997; Pelli 1997; Wilson et al. 2011) which allowed for randomisation of type and location of stimuli. Each stimulus consisted of a red RGB (255, 0, 0) bar measuring 6.5 cm in length and 1.5 cm in width (adjusted for the screen size), situated within an 8 cm diameter blue disk RGB (0, 0, 255), with a dominant wavelength at 450 nm, all set against a red background. These bars could be individually switched between horizontal and vertical orientations through key presses. The centre of the bars was aligned with the feeding stations (supplementary Fig. S1). In pilot studies, these colours were identified as the most easily discernible by bumblebees and allowed for effective video tracking of the bee against the background. Pilot studies also demonstrated no behavioural changes from bees while changing visual cues on the monitors whilst feeding. It is unlikely that the bee was able to see what was on the screen after landing due to the closeness to the screen, the attention focused towards the feeding station once the bee feeds, the need for bees to move in space to capture relevant visual information (Guiraud 2020), as well as the nature of the bee’s visual system. Bees possess a high temporal resolution 100 Hz for colour vision (Srinivasan and Lehrer 1985), and 200 Hz for achromatic patterns in movement (Srinivasan and Lehrer 1984) and a low spatial resolution 100 times less efficient than that of humans (Srinivasan et al. 1999). While we cannot exclude bees might sense some of the visual information while on the feeder, they are unlikely to see the patterns changing on-screen during the trials (for switching groups).

### Training

Pre-training with only blue stimuli (no bars) was used to train the bees to go to the different feeders. The eight blue disks were displayed against the red background, with each disk providing 10 μl of 30% sucrose solution (w/w). Once the bee successfully visited each of the feeder locations we began differential conditioning. In a training trial, four horizontal stimuli and four vertical stimuli were displayed on the screen. One type of stimulus (a horizontal or a vertical bar) was rewarded with 10μL of sucrose solution (50% w/w; CS +), while the other was punished with 10μL of saturated quinine solution (0.12% w/w; CS−). To ensure that bees relied solely on the visual cue for learning, the position of the stimuli was randomised between trials. Additionally, to prevent the potential influence of odour cues on the learning process, the entire arena and screen were cleaned with 70% ethanol in between each trial and test.

Bees were divided into four training groups: Constant Horizontal (CH), Constant Vertical (CV), Switching Horizontal (SH) and Switching Vertical (SV). In the “constant stimuli” groups (CH and CV), the orientation of the stimuli remained unchanged throughout each training trial, while in the “switching stimuli” groups (SH and SV) the orientation of the stimuli was switched between the bees’ arrival and departure (Fig. 1b). In the Constant Horizontal (CH) group (N = 13), bees were trained with the horizontal stimulus as rewarding (CS + , providing sucrose solution) and the vertical stimulus as punishing (CS− providing quinine solution). In the Constant Vertical (CV) group (N = 16), bees were trained to associate the vertical visual stimulus to the sucrose water (CS +) and the horizontal visual stimulus to saturated quinine solution (CS−). In the switching groups, the orientation of the CS + bar was changed as soon as bee landed on the feeder. This change was manually controlled by the experimenter using the keyboard (Fig. 1b). For example, when the bee alighted at a rewarded feeder with a vertical bar, the stimulus was switched to a horizontal bar so that the bee experienced different stimuli on arrival and upon departure from the rewarded stimuli (Fig. 1b). In the Switching Horizontal (SH) group (N = 10), bees were trained on the horizontal visual stimulus as rewarding (before landing) and the vertical bar as non-rewarding, but, as soon as the bee landed the horizontal stimulus was switched to the vertical stimulus. Finally, in the Switching Vertical (SV) group (N = 14) bees were trained on the vertical stimulus as rewarding (before landing) and the horizontal stimulus as non-rewarding, but, as soon as the bee finished feeding the vertical stimulus was replaced by the horizontal stimulus. Note that the CS− remained constant in the switching groups. Once the bee left the stimulus with no turn-back-and-look behaviour witnessed anymore, it was reset to its original condition prior to the bees next choice.

In each trial (defined as a bee’s visit to the arena, where it landed on different stimuli until satiated and subsequently returned to the nest), bees were free to land on multiple stimuli, feed, and revisit previously visited stimuli. To provide consistent reinforcement for each visit, the feeder of the visited stimulus was replenished with sucrose solution after the bee visited three out of the four rewarding feeding stations. During replenishment, the bee was briefly caught and placed in an opaque cup to prevent it from observing which platforms were refilled. Each individual bumblebee typically visited between three and ten feeders, with each landing counted as a visit. The training phase concluded when a bee exhibited ≥ 80% correct choices in the last twenty choices. It usually took between 5 and 20 trials to train a bee to reach the criterion and identify that one of the stimuli was a consistent indicator of reward.

### Testing

Following training, non-rewarded tests were performed replacing quinine or sugar with distilled water in the feeding stations. During tests, the number of correct and incorrect choices were recorded for 2 min. The refreshment trials with the training stimuli and the presence of sucrose reward and quinine solutions were interspersed (in a randomised fashion) among the non-rewarded tests to maintain the bees’ motivation. The bees had to reach  ≥ 80% correct choices in the refreshment trials before performing another test, with one to five inter-tests trials typically performed.

The three unrewarded tests are the learning test (the same stimuli from the training are shown to the bees), the conflict test and the generalisation test. Exploring the memory trace when bees are exposed to different visual stimuli before and after feeding, we created two tests. The conflict test that shows how bees respond in presence of intermediate visual stimuli and the original stimuli (is there any trace from the stimulus shown before and after feeding in the bees’ memory and a subsequent preference?), the generalisation test looks at the consequences of the generalisation of the CSs (do bees prefer angle of stimuli rather similar to the first CS seen upon arrival or the last CS seen upon departure?). In the conflict test, four stimuli with angles of 45°, 315° (two of each) along with four trained stimuli (horizontal and vertical, two of each) were presented to the bees to evaluate whether bees in the constant and switching stimuli groups used the pre-landing or post-landing visual features in their choices. In the generalisation test bees were presented with stimuli of the following angles: 22.5°, 67.5°; 112.5° 337.5°, two stimuli of each angle were presented (supplementary Fig S1). In both tests, locations of stimuli were randomly varied for each bee. This allowed us to assess if generalisation of the CS + differed between the switching and constant training groups.

### Statistical analysis

For each test, all contacts with feeders within a two-minute period were counted as choices. Statistical analysis was conducted using MATLAB (2021). To assess and compare the learning of bees during the training phase, we employed a Generalised Linear Mixed Model (GLMM). Bee performance through the training procedure was quantified as the percentage of correct choices in consecutive blocks of 10 visits. In the model, we included the blocks of 10 visits, the type of training groups (switching or consistent), the rewarding stimuli (horizontal and vertical), and the interaction between the choice block and training groups as explanatory variables. The model’s parameters were estimated using the Maximum Likelihood method within MATLAB.

To further analyse the performance of bees during the non-rewarding tests, we employed various statistical tests based on our hypothesis. The non-parametric Kruskal–Wallis H test was used to determine if there were statistically significant differences between the four groups of bees during tests. The Wilcoxon signed-ranked test was utilised to compare two related samples to assess whether their population mean ranks differ. Also, the Mann–Whitney U test also called Wilcoxon rank-sum test was used to compare two independent samples means, and test whether two sample means are equal or not. In all figures, means are presented along with standard errors of the mean.

## Results

### Effect of training treatment (constant versus switching) on learning

**Fig. 2:**
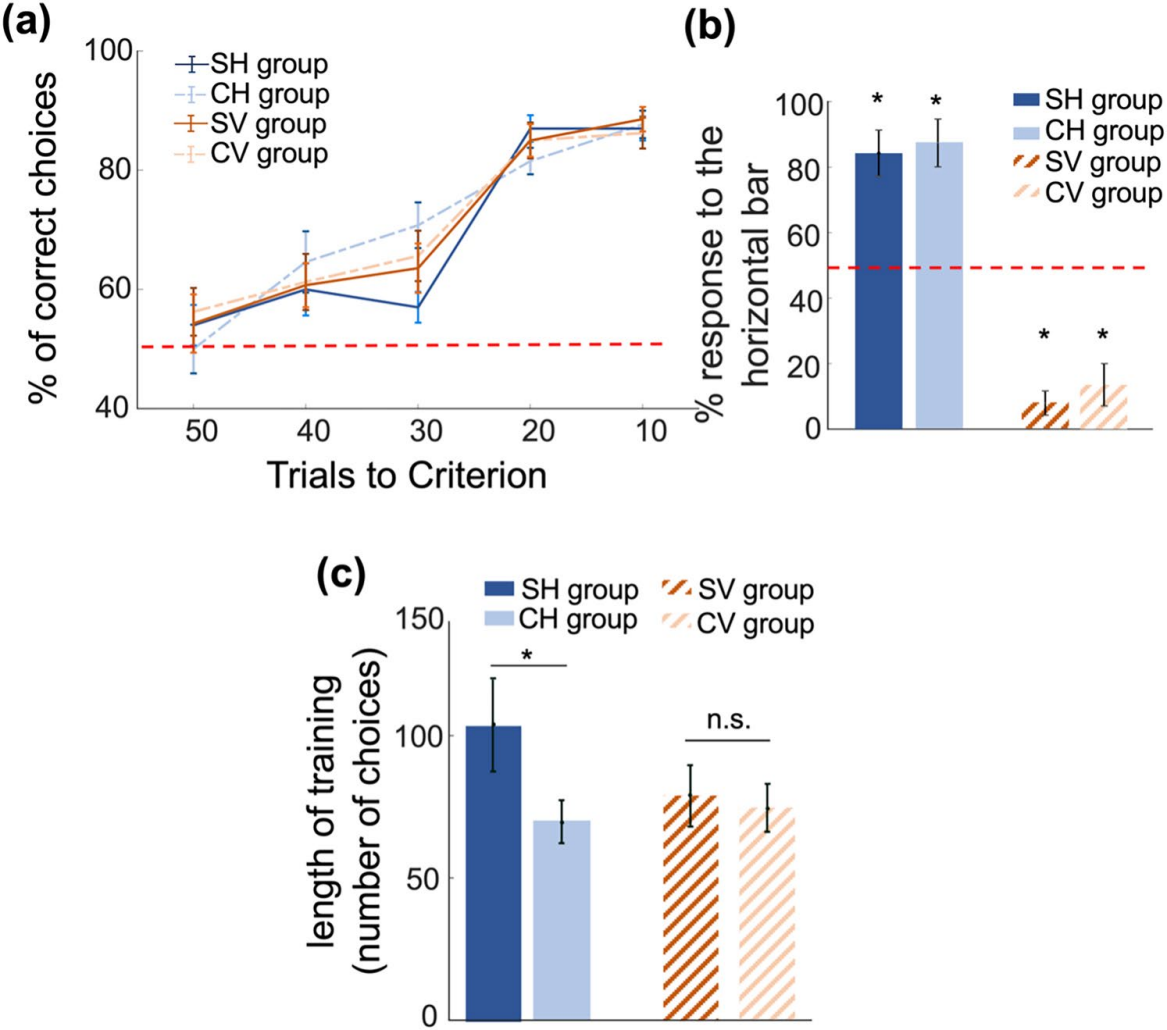
Bees’ length of training, training performance and choices during learning test. **a** Bees’ last 50 choices (means ± standard error; *P-value < 0.05). The red line represents chance level (50%). **b** Choices made by bees during the learning test (means ± standard error; *P-value < 0.05, different from chance). Bees trained with a horizontal CS + on approach (CH and SH) have a strong preference for the horizontal stimulus. Bees trained with a vertical CS + on approach (CV and SV) avoided horizontal stimulus (S3 Table). **c** Average length of time taken by bees to reach the threshold of 80% performance during the training phase (means ± standard error; *P-value < 0.05)

We used a Generalised Linear Mixed Model (GLMM: Formula: response ~ 1 + consecutive blocks of 10 choices + stimulus*protocol + (1 | bee_index). Model fit statistics: BIC = 1813.8, LogLikelihood = −888.75, Deviance = 1777.5) to explore factors influencing the proportion of correct choices made during training. The dependent variable was the number of correct choices from a block of 10 choices. Bee index was included in the model as a random factor (Table S1). Bees from all four groups learned the task (Fig. 2a) since their likelihood of selecting the rewarded stimuli increased over consecutive blocks of 10 choices: GLMM, P = 7.00e−07 (Table S1). In an unrewarded learning test, bees preferred the rewarded stimulus and avoided the punished stimulus (Fig. 2b, Table S1). Groups differed in their learning rate (GLMM P = 0.03, Table S1, Fig. 2a). Switching stimuli groups were slower than constant stimuli groups, with the greatest difference between the Switching Horizontal and Constant Horizontal stimuli groups.

Training of bees stopped when an individual made 80% (or more) correct choices within the last 20 choices, therefore the number of training choices differed for each bee. Bees in the Switching Horizontal stimuli group (in which the rewarded stimulus was horizontal on approach and vertical on departure from a stimulus) took more training choices to reach criterion than bees from the Constant Horizontal stimuli group (Mann–Whitney U test: U = 31.5, z = −2.047, P = 0.04, Fig. 2c). Bees from the Switching Vertical and Constant Vertical stimuli groups did not differ in number of training choices to reach criterion (Mann–Whitney U test CV versus SV U = 101, z = −0.436, P = 0.66, Fig. 2c). We compared performance of bees in the last 50 training choices until each bee reached the 80% correct criterion (Fig. 2a). Groups differed in their learning rate (GLMM P = 0.03, Table S1, Fig. 2a).

### Conflict test

**Fig. 3:**
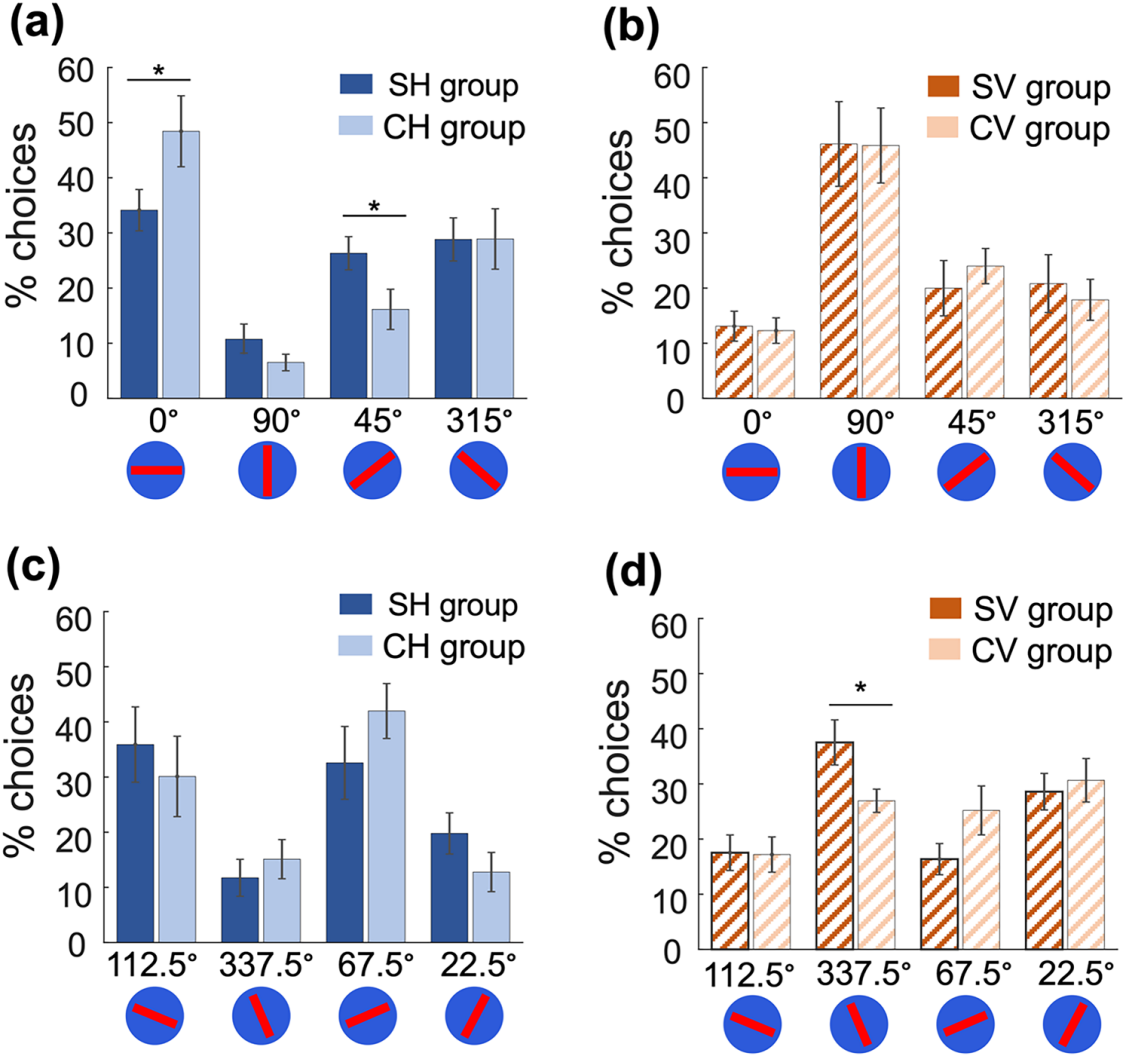
Bees’ performance in the conflict and generalisation tests. **a** and **b** Preference for each stimulus in the conflict test for bees trained with horizontal stimuli rewarded on approach (**a**) or vertical stimuli rewarded on approach (**b**) (means ± standard error, *P-value < 0.05). The SH stimuli group differed from the CH stimuli group (Table S3), but no differences were found between the SV and CV stimuli groups (Table S4). **c** and** d** Preference in the generalisation test for bees trained to horizontal (**c**; table S5) or vertical (**d**; table S6) rewarded stimuli on approach (means ± standard error; *P-value < 0.05). SV stimuli group shows a preference towards one angle in comparison with CV stimuli group (see result section)

In the unrewarded conflict test, bees were presented with horizontal and vertical bars as well as two intermediate stimuli of angled bars at 45° and 315° (Fig. 3a and b; Tables S3 and S4). Bees from all groups exhibited a preference for the stimulus they were trained on: vertical for the Constant Vertical and Switching Vertical stimuli groups (grouping the constant and switching vertical stimuli groups we tested if there were differences in terms of preference regarding the four options 0°, 45°, 90°, 315°: Kruskal–Wallis N = 112, H = 27.54, P < 0.001) and horizontal for the Constant Horizontal and Switching Horizontal stimuli groups (similarly to the vertical groups: Kruskal–Wallis N = 80, H = 29.08, P < 0.001). Bees in the Switching Horizontal stimuli group were more likely to choose one of the novel stimuli (Table S3: no difference between the choice of the horizontal bar and the novel stimuli, Wilcoxon-signed ranked test: P = NS for 45° and for 315° bars) and less likely to choose the horizontal stimulus than bees in the Constant Horizontal stimuli group (Table S3: only significant for 45° bar Wilcoxon-signed ranked test P = 0.02 and P = NS for 315° bar), but no difference in choices were seen between the Switching Vertical and Constant Vertical stimuli groups (Fig. 3b, Table S4 Wilcoxon-signed ranked test Responses between SV and CV groups P = NS for the horizontal bar, the vertical bar, for the 45° bar and for the 315° bar).

### Generalisation test

In the unrewarded generalisation test, bees were presented with two stimuli close to horizontal (67.5° and 112.5°) and two stimuli close to vertical (22.5° and 337.5°). Switching Horizontal and Constant Horizontal stimuli groups did not differ in their preference (Table S5, Wilcoxon signed-rank test difference between CH and SH P=NS for the 22.5° bar, the 67.5° bar, the 337.5° bar and the 112.5° bar), and both groups preferred the two stimuli close to horizontal (Table S5, Wilcoxon signed-rank test choice difference for the CH group: 112.5° vs 22.5° bars P = 0.005, 22.5° vs 67.5° bars P = 0.003; SH group 337.5° vs 112.5° bars P = 0.02 and 112.5° vs 22.5° bars P = 0.01). Switching Vertical stimuli group had a stronger preference for 337.5 than Constant Vertical stimuli group but no other differences were detected (Table S6, Wilcoxon signed-rank test difference CV vs SV for 112.5° bar preference P = 0.04).

Switching and constant stimuli groups showed minimal differences in their preferences in the generalisation test (Table S6, Wilcoxon signed-rank test for CV group differences between 337.5° and 112.5° bars P = 0.04, 337.5° vs 67.5° P = 0.03; for SV group differences between 337.5° and 67.5° P = 0.04, 112.5° vs 22.5° P = 0.004 and 22.5° vs. 67.5° P = 0.003). Interestingly, although bees exposed to the vertical bars in their forward inspection (CV and SV) were not as good as bees exposed to the horizontal bar in their forward inspection (CH group and SH group) in selecting the patterns with similar feature to the approach stimuli, the performance of bees were improved by experiencing different pattern in the post-landing inspection (i.e*.* SV group; Table S6).

## Discussion

Our study asked how bees deal with conflicting visual information when timing of learning is manipulated. While the visual task was learned quickly, we presented bees with conflicting information seen on arrival and departure from the feeder and this had minimal impact on either the rate of learning (Fig. 2a), or on the specificity of what had been learned. Learning of a horizontal CS + was more affected by conflicting information than a vertical CS+.

Our assay had features of a remaining visual engram characteristic of trace or backward conditioning. In backward conditioning, the unconditioned stimulus (US) is presented before the neutral stimulus (NS) (e.g. food before visual stimulus). It has been shown to be a less effective conditioning paradigm, however as it requires animals to link both information, a trace of the US and NS must persist. In trace conditioning, the neutral stimulus (NS) (visual stimulus) is presented before the unconditioned stimulus (US, food) but, with a temporal gap, in which the brain supposedly keeps a trace to bridge that gap and creates the association, the conditioned response. Since our stimuli would likely not been seen by bees when they were feeding, the CS would not overlap with the US. While classic associative learning conditioning happens when the CS precede the US, previous studies support backward conditioning (US before CS) (Spetch et al. 1981; Chang et al. 2004) and they imply that second-order conditioning, which describes a phenomenon when a conditioned stimulus (CS) acquires the ability to produce a conditioned response (CR) without being directly paired with an unconditioned stimulus (US), reveal that time is part of what is learnt (Molet and Miller 2014). Such mechanism hasn’t been studied in bees and our work could help better understand these mechanisms. Lehrer was the first to show bees learn information seen on both arrival and departure from a sucrose solution feeder (1991, 1993). She found that if bees were presented with different stimuli on arrival and departure from a feeder their learning rate slowed. While there was evidence bees could learn a stimulus seen on departing a feeder, they showed a prioritisation of the stimulus seen before feeding (Bitterman and Couvillon 1991; Lehrer 1993). Our work differs from Lehrer (1993) in that she asked if bees could learn two CS + (one on approach, one during departure). This can be solved by using an additive process or visual generalisation. She presented bees with two different stimuli on arrival and departure (essentially two CS +), while we introduced a conflict, using a discriminant learning paradigm and presented some bees with a conflict situation (CS + seen on arrival, CS− seen on departure). In this case, we saw no reduction in learning rate when compared to learning a consistent CS + for a vertical CS + stimulus, and only a minor reduction in learning rate for learning a horizontal CS + . Similarly, in generalisation tests the conflicting information had minimal impact. It is clear, therefore, that, when presented with the CS + flipped to the CS− on departure from a sucrose feeder bumblebees did not generalise between the two stimuli, nor was there interference between the two stimuli. Bees in the switching groups appear to prioritise the relevant CS + information and entirely disregard the conflicting CS− information, but we may not need to invoke cognitive concepts such as “prioritisation” to explain our findings.

In our study, we use the terms CS + and CS− in a way that departs from conventional classical conditioning paradigms to capture the nuances of backward and trace conditioning in bees. For switching groups, our experimental paradigm coins the correct arrival stimulus as CS + (paired with a sucrose solution) while during departure it transforms as the stimulus encountered as CS−. This design allowed us to explore how bees process visual stimuli encountered before and after feeding. We recognise that, in this case, our use of CS + and CS− terminology differs from typical classical conditioning, where CS + generally signals an appetitive stimulus and CS− an aversive or neutral one. However, this choice reflects the unique nature of backward conditioning in bees, as studied by Lehrer (1991, 1993) and others, and aligns with trace conditioning, which requires animals to retain a temporal memory of the reinforcement beyond the feeding period. This conditioning paradigm is less frequently studied, particularly in bees, and our findings provide new insights into these mechanisms. Additionally, bees might investigate the stimuli at different time length during arrival or departure, potentially involving temporal consistency/contingency in their visual learning. Further analysis of bees’ scanning behaviours is needed to evaluate this aspect of the temporal investigation. Our findings suggest that bees may employ more complex mechanisms, beyond straightforward temporal or spatial contiguity, to prioritise CS + information. These insights emphasise the need for a deep understanding of bees’ learning processes in paradigms involving backward and trace conditioning, shedding light on the intricate nature of associative learning in insects.

The most plausible anatomical locus for the associative learning phenomena studied here are the mushroom bodies (Barth and Heisenberg 1997; Li et al. 2017). The Kenyon cells of the mushroom bodies receive processed sensory input, and output from premotor regions (Mobbs 1982; Fahrbach 2006). There is experience-dependent neuroplasticity at both the input and output of the Kenyon cells that is sensitive to neurochemicals released in response to appetitive or aversive reinforcers (Barnstedt et al. 2016). It is theoretically possible for the mushroom body to support an enduring trace of neural activation for a short period of time (Menzel 2001; Menzel and Giurfa 2001). The Kenyon cells have a prolonged accommodation property (Strausfeld et al. 2009), and in *Drosophila*, recurrent connections have been detected between Kenyon cells (Dylla et al. 2013; Lyutova et al. 2019; Chandra et al. 2010; Aso et al. 2014; Bennett et al. 2021). These could, in theory, support a reverberation of neural activity in the Kenyon cell populations. Either or both mechanisms could maintain a trace of neural activity that persists beyond the presentation of a stimulus. This could support elementary forms of trace conditioning and the phenomena we are seeing here.

Here, the CS is seen after the US, due to the turn-back-and-look behaviour influenced bees’ preferences during the conflict test (preference of 45° bars over CS−). This finding could be supported by a form of secondary reinforcement (last stimuli seen associated with the food reward) or latent learning (no explicit reinforcer) (Menzel 2001). Both secondary reinforcement and latent learning are believed to involve the mushroom bodies in conjunction with the spatial systems of the lateral accessory lobes (Wystrach 2023).

If mushroom bodies are involved in learning the stimuli seen both before and after feeding, how is it that learning performance is largely unchanged even if this information conflicts? In both the conflict test and the generalisation test, bees exhibited a preference towards the first CS + seen (for switching groups). During the conflict test, the first choice for all groups was the 1st CS + seen (horizontal bar for CH and SH group and the vertical bar for CV and SV group) and for the generalisation test the angle closest to the original CS + was favoured as well albeit a few group differences. In terms of the robustness of bees to learning conflicted information, here we should consider the mechanisms of decision making in bees as well as the learning mechanisms. Ultimately, the outcome of learning is to influence a decision of whether a bee should land at a feeder marked by a horizontal or vertical stimulus. The mushroom body alone is not a decision maker (Galizia 2014; Bazhenov et al. 2013; Huerta et al. 2004, 2009). It can perhaps best be thought of a as a classifier—learning to associate presented stimuli with different outcomes which are conveyed by mushroom body output neurons to premotor regions (Galizia 2014; MaBouDi et al. 2023). The punished stimuli (fixed for CS− seen both on arrival and departure) were consistent in all groups (while for switching groups the CS + was changed from CS + before feeding to CS− after feeding) therefore the rate of learning to avoid the CS− would be the same in all groups. In both the switching and constant groups, the CS + was seen on approach to the feeder, therefore in all groups the CS + was reinforced for approach behaviour only, whereas the CS− stimulus would be reinforced for avoidance of punished stimuli in all groups and departure from the CS + in the switching groups. If we consider the mushroom body as classifying stimuli by behavioural response, this alone is sufficient to resolve any conflicting information associated with a feeder. In our paradigm, the CS + was only associated with approach responses, regardless of training groups.

In this experiment, learning of a horizontal CS + was more disrupted by the switching manipulation than learning of a vertical CS + . The horizon has a natural value for navigational animals (Gould 1998) but when it comes to stimuli, other explanation might be possible. While there are other reports of insects responding differently to vertical and horizontal stimuli or learning them at different rates (Srinivasan et al. 1999; Wang et al. 2014; Wolf et al. 2015). It is not clear why it is happening. Previous literature suggests that the ecological or functional implication of spatial positioning of flowers (horizontal or vertical) might be at play. Flowers can be arranged both horizontally in meadows or vertically in inflorescences or bushes. However, pollinators’ preference (bumblebee here) might lean towards horizontal arrangements of flowers (meadows) which could explain why the manipulation of horizontal stimuli was more impacted than for the vertical stimuli. Previous work has shown that the horizontal distribution of flowers (meadows) and its subsequent foraging’s patterns maximise nectar income (Pyke 1978; Heinrich 1979a, b; Dreisig 1995; Keasar et al. 1996; Chittka et al. 1997; Keasar 2000; Cresswell and Osborne 2004; Wolf and Moritz 2008; Lihoreau et al. 2012) contrasting with bumblebees’ slower foraging pattern (bottom up) when it comes to vertical inflorescences (e.g. Pyke 1979; Waddington and Heinrich 1979). However, these studies did not directly compare horizontally *versus* vertically arranged flowers. Moreover, perceptual limitations from functional differences from the bees’ eye with regional morphological specialization at the ommatidia level which affect their field of view could facilitate (or not) edge detection (and spatial detection; (Wehner 1972; Chittka et al. 1988; Lehrer 1999; Taylor et al. 2019). Additionally, both honeybees and bumblebees show a pronounced dorso-ventral segregation of different neuronal wiring happening at various levels in the visual system (Hertel 1980; Ehmer and Gronenberg 2002; Paulk et al. 2008; Mota et al. 2011) adding another level of complexity when it comes to edge detection and processing, as vertical and horizontal cues may be processed differently depending on visual andtemporal constraints (Giurfa et al. 1999; Giurfa 2004; Stach and Giurfa 2005; Dyer et al. 2008; Dyer 2012; Dyer and Griffiths 2012; Morawetz and Spaethe 2012; Morawetz et al. 2013). All these factors could feed into why there are differences in processing vertical and horizontal visual cues. Finally, Wolf et al. (2015) suggests that attentional processes born from experience could offer a parsimonious explanation regarding bumblebees’ preferences towards horizontally oriented meadows. While horizontal meadows usually offer a range of flower types and species, bees can easily avoid revisiting flowers, whereas in vertical inflorescences, bushes or tree only one single flower type is offered. Hence, we could understand that in such instance a higher cognitive demand is necessary to remember what flower was visited and when, increasing foraging time and affecting efficiency. For single-foragers bees such as *Bombus terrestris* this could impact the fitness of the colony as their survival depends upon a handful of foragers at each time contrarily to honeybees.

In summary, our study demonstrates remarkable speed and proficiency for bumble bees learning to overcome a seemingly conflicting paradigm. Their learning was rapid, specific and largely unaffected if the CS + feeder was linked with conflicting information. Our study speaks to the remarkable efficacy of the bee brain for learning food related stimuli.

## Supplementary Information

Below is the link to the electronic supplementary material.

Supplementary file1 (DOCX 623 kb)

## Data Availability

Available upon demand.
