# Supplementary material for: How bumblebees manage conflicting information seen on arrival and departure from flowers
Source: Anim Cogn. 2025 Feb 5;28(1):11. doi: 10.1007/s10071-024-01926-x (PMC11799123; doi:10.1007/s10071-024-01926-x)
Supplement: Supplementary file 1 — Supplementary file1 (DOCX 623 kb) [file 10071_2024_1926_MOESM1_ESM.docx]

# Supplementary material

**
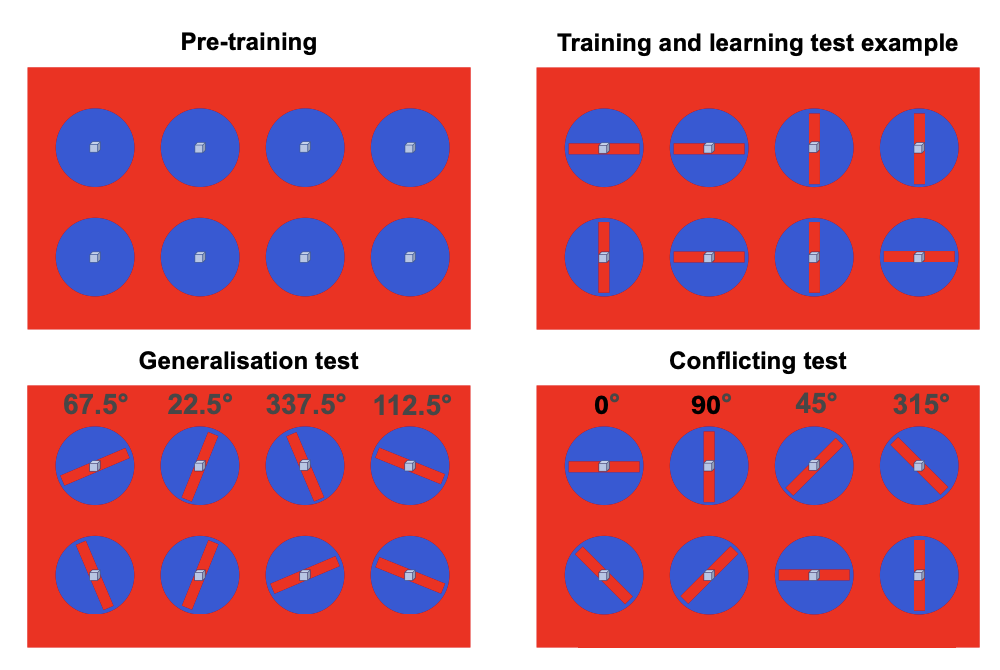
**

**Fig S1. Training and testing.** The pre-training with blue stimuli was used to train the bees to go to the different feeders. In the training, four similar positively reinforced stimuli (CS+) and four similar negatively reinforced stimuli (CS-) were shown and randomly interspersed between trials. The learning test was similar to the training with differently positioned CS+ and CS-. The generalisation test showed different angles than the training (22.5°, 67.5°, 112.5°, 337.5°). The conflict test showed CS+ and CS- as well as angles in between the two CS (45° and 315°).


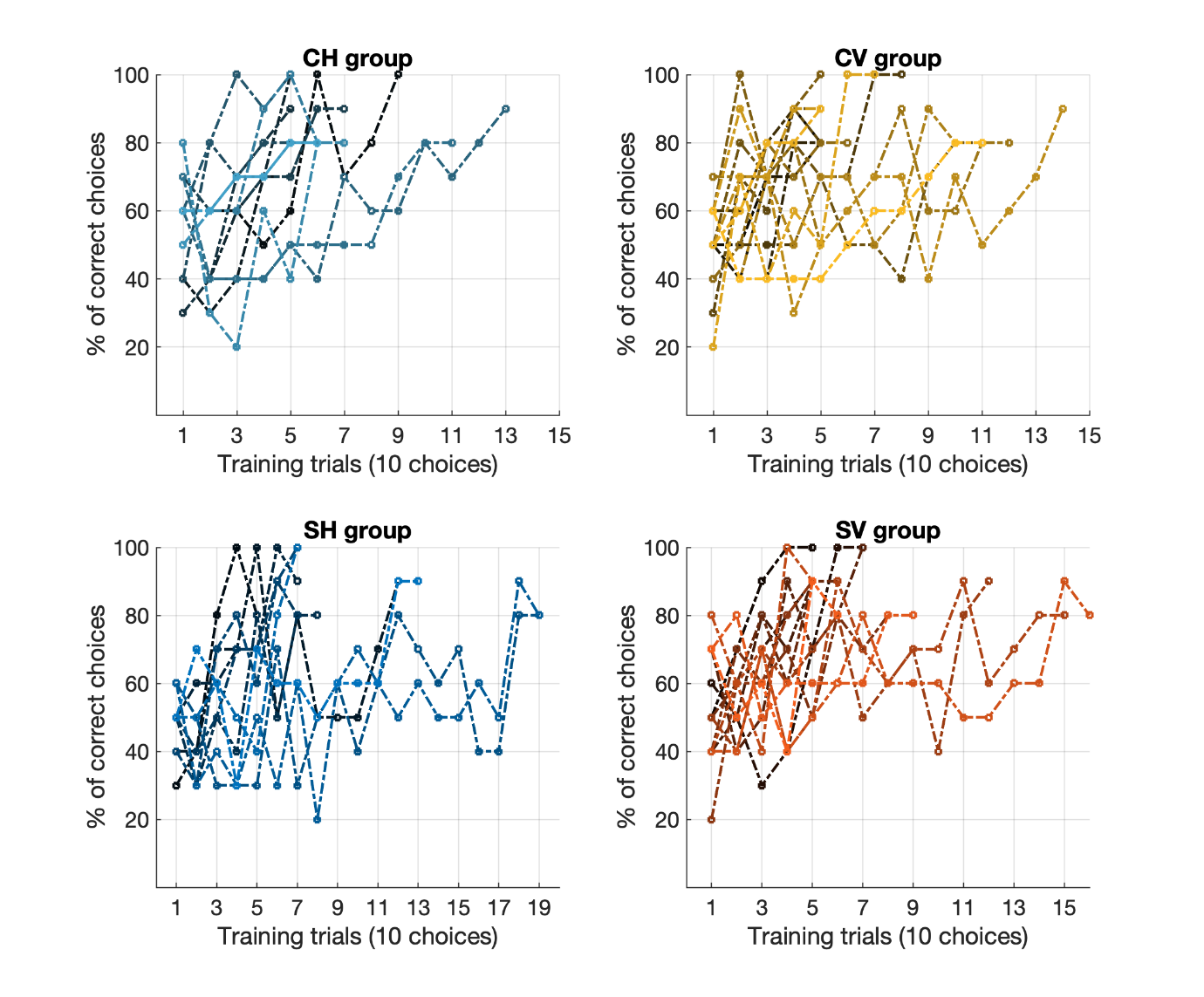


**Fig S2. Training trials for each group (all bees).** Individual data shown *per* group CH, CV, SH and SV. Bees from CH group reached criterion quicker than bees from SH, whereas CV and SV groups performed in a more similar fashion regarding reaching criterion.

**Table S1. Summary of the Generalised Linear Mixed Model (GLMM) examining factors in relation to proportion of rewarded choices during the training.** Formula: response ~ 1 + consecutive blocks of 10 choices + stimulus*protocol + (1 | bee_index). Model fit statistics: BIC= 1813.8, LogLikelihood=-888.75, Deviance=1777.5.

| \| **Fixed factors** \| **Estimate** \| **SE** \| **tStat** \| **DF** \| **P-value** \| **Lower** \| **Upper** \| \| --- \| --- \| --- \| --- \| --- \| --- \| --- \| --- \| \| **Intercept** \| 2.0562 \| 0.21472 \| 9.5763 \| 420 \| 8.84e-20 \| 1.63 \| 2.47 \| \| **Consecutive blocks of 10 choices** \| 0.02 \| 0.005 \| 5.037 \| 420 \| **7.00e-07** \| 0.01 \| 0.03 \| \| **Stimulus (horizontal and vertical** \| -0.14 \| 0.13 \| -1.06 \| 420 \| 0.28 \| -0.39 \| 0.11 \| \| **Protocol (control *versus* switching** \| -0.28 \| 0.13 \| -2.08 \| 420 \| **0.03** \| -0.56 \| -0.01 \| \| **Stimulus : protocol** \| 0.13 \| 0.08 \| 1.64 \| 420 \| 0.10 \| -0.02 \| 0.30 \| |
| --- | --- | --- | --- | --- | --- | --- | --- | --- | --- | --- | --- | --- | --- | --- | --- | --- | --- | --- | --- | --- | --- | --- | --- | --- | --- | --- | --- | --- | --- | --- | --- | --- | --- | --- | --- | --- | --- | --- | --- | --- | --- | --- | --- | --- | --- | --- | --- | --- |

**Table S2. Statistical analysis for Fig 2b**

| **Variable** | **Statical test** | **Statistical value** | **Number bees** | **p-value** |
| --- | --- | --- | --- | --- |
| Response to horizontal bar in SH | Wilcoxon rank sum | 2.68 | 5 | **0.007** |
| Response to horizontal bar in CH | Wilcoxon rank sum | 2.68 | 5 | **0.007** |
| Response to horizontal bar in SV | Wilcoxon rank sum | -3.01 | 6 | **0.002** |
| Response to horizontal bar in CV | Wilcoxon rank sum | -2.68 | 5 | **0.007** |

**Table S3. Statistical analysis for Fig 3a**

| **Variable** | **Statical test** | **Statistical value** | **Number bees** | **P-value** |
| --- | --- | --- | --- | --- |
| Bee responses to 45°-bar vs. to horizontal bar in the group CH | Wilcoxon signed rank | -2.80 | 10 | **0.005** |
| Bee responses to 45°-bar vs. to vertical bar in the group CH | Wilcoxon signed rank | 1.82 | 10 | 0.06 |
| Bee responses to 315°-bar vs. to horizontal bar in the group CH | Wilcoxon signed rank | -1.52 | 10 | 0.12 |
| Bee responses to 315°-bar vs. to vertical bar in the group CH | Wilcoxon signed rank | 2.66 | 10 | **0.007** |
| Bee responses to 45°-bar vs. to horizontal bar in the group SH | Wilcoxon signed rank | -1.12 | 10 | 0.26 |
| Bee responses to 45°-bar vs. to vertical bar in the group SH | Wilcoxon signed rank | 2.52 | 10 | **0.01** |
| Bee responses to 315°-bar vs. to horizontal bar in the group SH | Wilcoxon signed rank | -0.33 | 10 | 0.73 |
| Bee responses to 315°-bar vs. to vertical bar in the group SH | Wilcoxon signed rank | -2.49 | 10 | **0.01** |
| Bee responses to the horizontal bar in group SH vs group CH | Wilcoxon rank sum | -1.96 | 10, 10 | **0.04** |
| Bee responses to the vertical bar in group SH vs group CH | Wilcoxon rank sum | 0.95 | 10, 10 | 0.33 |
| Bee responses to 45°-bar in group SH vs group CH | Wilcoxon rank sum | 2.19 | 10, 10 | **0.02** |
| Bee responses to 315°-bar in group SH vs group CH | Wilcoxon rank sum | 0.00 | 10, 10 | 1.0 |

**Table S4. Statistical analysis for Fig 3b**

| **Variable** | **Statical test** | **Statistical value** | **Number bees** | **P-value** |
| --- | --- | --- | --- | --- |
| Bee responses to 45°-bar vs. to horizontal bar in the group CV | Wilcoxon signed rank | 2.93 | 14 | **0.003** |
| Bee responses to 45°-bar vs. to vertical bar in the group CV | Wilcoxon signed rank | -1.91 | 14 | 0.055 |
| Bee responses to 315°-bar vs. to horizontal bar in the group CV | Wilcoxon signed rank | 1.47 | 14 | 0.13 |
| Bee responses to 315°-bar vs. to vertical bar in the group CV | Wilcoxon signed rank | -2.37 | 14 | **0.01** |
| Bee responses to 45°-bar vs. to horizontal bar in the group SV | Wilcoxon signed rank | 1.38 | 14 | 0.16 |
| Bee responses to 45°-bar vs. to vertical bar in the group SV | Wilcoxon signed rank | -2.20 | 14 | **0.02** |
| Bee responses to 315°-bar vs. to horizontal bar in the group SV | Wilcoxon signed rank | 1.53 | 14 | 0.12 |
| Bee responses to 315°-bar vs. to vertical bar in the group SV | Wilcoxon signed rank | -1.88 | 14 | 0.059 |
| Bee responses to the horizontal bar in group SV vs group CV | Wilcoxon rank sum | 0.11 | 14, 14 | 0.90 |
| Bee responses to the vertical bar in group SV vs group CV | Wilcoxon rank sum | 0.27 | 14, 14 | 0.78 |
| Bee responses to 45°-bar in group SV vs group CV | Wilcoxon rank sum | -1.38 | 14, 14 | 0.16 |
| Bee responses to 315°-bar in group SV vs group CV | Wilcoxon rank sum | 0.36 | 14, 14 | 0.71 |

**Table S5. Statistical analysis for Fig 3c**

| **Variable**  Bee responses to | **Statical test** | **Statistical value** | **Number bees** | **P-value** |
| --- | --- | --- | --- | --- |
| 337.5°bar vs. 112.5°bar in the group CH | Wilcoxon signed rank | 1.60 | 12 | 0.10 |
| 337.5°bar vs. 22.5°bar in the group CH | Wilcoxon signed rank | -1.88 | 12 | 0.059 |
| 337.5°bar vs. 67.5°bar in the group CH | Wilcoxon signed rank | 1.76 | 12 | 0.07 |
| 112.5°bar vs. 22.5°bar in the group CH | Wilcoxon signed rank | -2.80 | 12 | **0.005** |
| 112.5°bar vs. 67.5°bar in the group CH | Wilcoxon signed rank | 0.33 | 12 | 0.73 |
| 22.5°bar vs. 67.5°bar in the group CH | Wilcoxon signed rank | 2.93 | 12 | **.003** |
| 337.5°bar vs. 112.5°bar in the group SH | Wilcoxon signed rank | 2.19 | 10 | **0.02** |
| 337.5°bar vs. 22.5°bar in the group SH | Wilcoxon signed rank | -0.15 | 10 | 0.87 |
| 337.5°bar vs. 67.5°bar in the group SH | Wilcoxon signed rank | 1.89 | 10 | 0.057 |
| 112.5°bar vs. 22.5°bar in the group SH | Wilcoxon signed rank | -2.38 | 10 | **0.01** |
| 112.5°bar vs. 67.5°bar in the group SH | Wilcoxon signed rank | -1.68 | 10 | 0.09 |
| 22.5°bar vs. 67.5°bar in the group SH | Wilcoxon signed rank | 1.17 | 10 | 0.24 |
| Bee responses to 337.5°bar between CH and SH | Wilcoxon rank sum | 0.85 | 10, 12 | 0.39 |
| Bee responses to 112.5°bar between CH and SH | Wilcoxon rank sum | -0.73 | 10, 12 | 0.46 |
| Bee responses to 22.5°bar between CH and SH | Wilcoxon rank sum | -1.12 | 1,0 12 | 0.25 |
| Bee responses to 67.5°bar between CH and SH | Wilcoxon rank sum | 1.36 | 10, 12 | 0.17 |

**Table S6. Statistical analysis for Fig 3d**

| **Variable**  Bee responses to | **Statical test** | **Statistical value** | **Number bees** | **P-value** |
| --- | --- | --- | --- | --- |
| 337.5°bar vs. 112.5°bar in the group CV | Wilcoxon signed rank | -1.97 | 16 | **0.04** |
| 337.5°bar vs. 22.5°bar in the group CV | Wilcoxon signed rank | -1.29 | 16 | 0.19 |
| 337.5°bar vs. 67.5°bar in the group CV | Wilcoxon signed rank | -2.07 | 16 | **0.03** |
| 112.5°bar vs. 22.5°bar in the group CV | Wilcoxon signed rank | 0.26 | 16 | 0.78 |
| 112.5°bar vs. 67.5°bar in the group CV | Wilcoxon signed rank | -0.59 | 16 | 0.55 |
| 22.5°bar vs. 67.5°bar in the group CV | Wilcoxon signed rank | -0.80 | 16 | 0.42 |
| 337.5°bar vs. 112.5°bar in the group SV | Wilcoxon signed rank | -2.58 | 14 | **0.009** |
| 337.5°bar vs. 22.5°bar in the group SV | Wilcoxon signed rank | 0.08 | 14 | 0.92 |
| 337.5°bar vs. 67.5°bar in the group SV | Wilcoxon signed rank | -2.03 | 14 | **0.04** |
| 112.5°bar vs. 22.5°bar in the group SV | Wilcoxon signed rank | 2.83 | 14 | **0.004** |
| 112.5°bar vs. 67.5°bar in the group SV | Wilcoxon signed rank | 1.09 | 14 | 0.27 |
| 22.5°bar vs. 67.5°bar in the group SV | Wilcoxon signed rank | -2.15 | 16 | **0.03** |
| Bee responses to 337.5°bar between CV and SV | Wilcoxon rank sum | 0.0 | 16, 14 | **0.04** |
| Bee responses to 112.5°bar between CV and SV | Wilcoxon rank sum | 2.01 | 16, 14 | 1.0 |
| Bee responses to 22.5°bar between CV and SV | Wilcoxon rank sum | -1.37 | 16, 14 | 0.16 |
| Bee responses to 67.5°bar between CV and SV | Wilcoxon rank sum | 1.66 | 16, 14 | 0.86 |
